# A multicenter trial of the efficacy and safety of tigecycline versus imipenem/cilastatin in patients with complicated intra-abdominal infections [Study ID Numbers: 3074A1-301-WW; ClinicalTrials.gov Identifier: NCT00081744]

**DOI:** 10.1186/1471-2334-5-88

**Published:** 2005-10-19

**Authors:** María E Oliva, Arcot Rekha, Albert Yellin, Jacyr Pasternak, Maria Campos, Gilbert M Rose, Timothy Babinchak, Evelyn J Ellis-Grosse, Evan Loh, the 301 Study Group

**Affiliations:** 1 Hospital San Martin, Provincia de Entre Rios, Argentina; 2 Sri Ramachandra Medical College and Research Institute, Tamil Nadu, India; 3 LAC-USC Medical Center, Los Angeles, California. USA; 4 Real E Benemérita Sociedade Portuguesa de Beneficéncia, Hospital Säo Jaoquim, São Paulo/SP, Brazil; 5 Hospital de Urgencia Asistencia Publica, Santiago, Chile; 6 Clinical Research Group, Wyeth Research, Collegeville, Pennsylvania, USA

## Abstract

**Background:**

Complicated intra-abdominal infections (cIAI) remain challenging to treat because of their polymicrobial etiology including multi-drug resistant bacteria. The efficacy and safety of tigecycline, an expanded broad-spectrum glycylcycline antibiotic, was compared with imipenem/cilastatin (IMI/CIS) in patients with cIAI.

**Methods:**

A prospective, double-blind, multinational trial was conducted in which patients with cIAI randomly received intravenous (IV) tigecycline (100 mg initial dose, then 50 mg every 12 hours [q12h]) or IV IMI/CIS (500/500 mg q6h or adjusted for renal dysfunction) for 5 to14 days. Clinical response at the test-of-cure (TOC) visit (14–35 days after therapy) for microbiologically evaluable (ME) and microbiological modified intent-to-treat (m-mITT) populations were the co-primary efficacy endpoint populations.

**Results:**

A total of 825 patients received ≥ 1 dose of study drug. The primary diagnoses for the ME group were complicated appendicitis (59%), and intestinal (8.8%) and gastric/duodenal perforations (4.6%). For the ME group, clinical cure rates at TOC were 80.6% (199/247) for tigecycline versus 82.4% (210/255) for IMI/CIS (95% CI -8.4, 5.1 for non-inferiority tigecycline versus IMI/CIS). Corresponding clinical cure rates within the m-mITT population were 73.5% (227/309) for tigecycline versus 78.2% (244/312) for IMI/CIS (95% CI -11.0, 2.5). Nausea (31.0% tigecycline, 24.8% IMI/CIS [*P *= 0.052]), vomiting (25.7% tigecycline, 19.4% IMI/CIS [*P *= 0.037]), and diarrhea (21.3% tigecycline, 18.9% IMI/CIS [*P *= 0.435]) were the most frequently reported adverse events.

**Conclusion:**

This study demonstrates that tigecycline is as efficacious as imipenem/cilastatin in the treatment of patients with cIAI.

## Background

Complicated intra-abdominal infections are characterized as local or systemic infections secondary to a physical perforation in the gastrointestinal tract or via a necrotic gut wall into the peritoneal space, leading to abscess formation or peritonitis [1]. These infections require a combination of appropriate and timely surgical source control and broad spectrum antimicrobial therapy for optimal outcome. Nearly all intra-abdominal infections are caused by multiple microorganisms resident in the gastrointestinal tract; these include aerobes and facultative and obligate anaerobes [2], with Enterobacteriaceae (eg, *Escherichia coli*) isolated most frequently [1,3]. Although isolation of enterococci from an intra-abdominal source were once suggestive of normal flora, these bacteria are now recognized as true pathogens, with upwards of one third of intra-abdominal cultures yielding enterococci [2]. In fact, the isolation of *Enterococcus *spp. from an intra-abdominal focus of infection has been linked with treatment failure [4].

Treatment of complicated intra-abdominal infections remains a challenge, primarily because of their polymicrobial etiology coupled with the high risk of complications and death. Because frequently recovered isolates may possess multiple resistance factors (eg, extended spectrum beta-lactamases [ESBLs]) that express antimicrobial resistance, empiric antimicrobial therapy should have anticipated activity against these difficult-to-treat isolates [1,5]. As such, combination antibiotic therapy has often been a standard of care for treatment of these infections [1]. The recent 2003 guidelines of the Infectious Diseases Society of America (IDSA) advocates broad-spectrum single or combination therapy (eg, carbapenem or piperacillin/tazobactam monotherapy, third- or fourth-generation cephalosporins or fluoroquinolones plus metronidazole) for high-risk patients with severe or postoperative nosocomial intra-abdominal infections wherein polymicrobial infection and/or resistant flora are more prevalent [1]. When very resistant bacteria are suspected (eg, vancomycin-resistant *Enterococcus*, methicillin-resistant *Staphylococcus aureus*, *Pseudomonas aeruginosa*), however, a complex multidrug regimen is recommended [1]. The initial selection of antimicrobial therapy for treatment of intra-abdominal infections is extremely important because inappropriate empiric antimicrobial therapy has been associated with delayed clinical resolution, increased length of hospital stay, and an increased risk of mortality [6,7]. Adequate surgical source control is also an important determinant of outcome; insufficient drainage and repair may compromise the effectiveness of antibiotic therapy [1].

Tigecycline is a novel, first-in-class, glycylcycline antibiotic with expanded broad-spectrum wide in vitro activity against the microorganisms commonly encountered in intra-abdominal infections. Specifically, tigecycline's spectrum of in vitro activity includes aerobic and facultative gram-positive and gram-negative bacteria and anaerobic bacteria [8-11]. Tigecycline also provides in vitro activity against antibiotic-resistant bacteria such as vancomycin-resistant *Enterococcus faecalis *and *E. faecium*, ESBL-producing enteric gram-negative bacteria, and methicillin-resistant *S. aureus *[8-16]. The primary objective of this multicenter trial was to evaluate the efficacy and safety of tigecycline monotherapy compared with imipenem/cilastatin in the treatment of hospitalized adult patients with complicated intra-abdominal infections. A second goal of the study was to evaluate the in vitro susceptibility of tigecycline against common bacteria implicated as causes of intra-abdominal infection.

## Methods

### Study design and enrollment criteria

This was a phase 3, multicenter, double-blind (third-party unblinded) trial of adult patients who were candidates for or had undergone a laparotomy, laparoscopy, or percutaneous drainage of an intra-abdominal abscess and had a known or suspected diagnosis of complicated intra-abdominal infection. All patients were hospitalized at the time of study entry. Before screening of the first patient, the protocol was reviewed and approved by the institutional review board or ethical review committee at each participating center. Written informed consent was obtained from each patient or his or her legal representative before the start of any study procedures. The trial was conducted in accordance with the Declaration of Helsinki.

#### Inclusion criteria

Men and women were eligible for inclusion if they were 18 years of age or older and required a surgical procedure for a complicated intra-abdominal infection. Complicated intra-abdominal infections included conditions such as an intra-abdominal abscess (including liver and spleen) that developed in a postsurgical patient after receiving standard antibacterial therapy (ie, at least 48 hours, but note more than 5 days of antibiotics); appendicitis complicated by perforation and/or a periappendiceal abscess; perforated diverticulitis complicated by abscess formation or fecal contamination; complicated cholecystitis with evidence of perforation, empyema, or gangrene; perforation of a gastric or duodenal ulcer with symptoms exceeding 24 hours; purulent peritonitis or peritonitis associated with fecal contamination; or perforation of the large or small intestine with abscess or fecal contamination. In addition, patients could not have received more than 1 dose of an antibiotic (single broad-spectrum agent or 1 dose of each antibiotic in a combination regimen such as metronidazole, ampicillin, gentamicin) after the baseline intra-abdominal culture was obtained from the infected site.

#### Exclusion criteria

Patients were not allowed to participate if they had any concomitant condition that precluded evaluation of a response or made it unlikely that the planned course of therapy could be completed. Other primary reasons for ineligibility included the following: preoperative suspicion of a diagnosis of spontaneous bacterial peritonitis, simple cholecystitis, gangrenous cholecystitis without rupture, simple appendicitis, acute suppurative cholangitis, pancreatic abscess, or infected necrotizing pancreatitis; Acute Physiologic and Chronic Health Evaluation (APACHE) II score greater than 30; active or treated leukemia or systemic malignancy within the prior 3 months or metastatic malignancy to the abdomen within the prior 6 months; known acquired immunodeficiency syndrome (AIDS); presence of any uncontrolled central nervous system disease; pregnant or breastfeeding women; known or suspected hypersensitivity to either study drug or to related compounds; concomitant ganciclovir therapy; significant hepatic disease (ie, aspartate aminotransferase [AST] or alanine aminotransferase [ALT] level > 10 times the upper limit of normal [ULN] or total bilirubin value > 3 times the ULN) or acute hepatic failure or acute decompensation of chronic hepatic failure; significant renal disease (ie, calculated creatinine clearance < 41 mL/min/1.73 m^2 ^after adequate hydration); neutropenia with absolute neutrophil count < 1000/mm^3^, with counts as low as 500/mm^3 ^permitted if due to the acute infectious process; current intra-abdominal infection known to be caused by one or more bacterial isolates not susceptible to either of the study drugs (eg, *P. aeruginosa, Proteus mirabilis*); surgical procedure requiring that fascia or deep muscular layers be left open or expectation of planned abdominal re-exploration either in or out of the operating room; and administration of intraoperative antibacterial irrigants or peritoneal antibacterial agents (eg, irrigants, antibiotic-impregnated sponges). Any patient requiring additional systemic antibacterial therapy, for any reason, was not allowed to participate in the trial.

### Antimicrobial regimens

Patients were stratified at randomization into 2 groups based on their scores on APACHE II: ≤15, or >15 but <31. Using a 1:1 ratio, patients were randomly assigned to receive either tigecycline (initial 100-mg dose given by intravenous [IV] infusion over a 30-minute period, followed by 50 mg IV every 12 hours) or IV imipenem/cilastatin (500 mg/500 mg every 6 hours or dose-adjusted based on weight and creatinine clearance). Patients randomized to tigecycline received a 100 mL normal saline intravenous infusion 6 hours after active drug each day in order to maintain the blind. Unless the patient was a clinical failure (see definition below), the duration of study drug therapy ranged from 5 to 14 days.

Study drug was administered only when there was a strong suspicion (ie, elevated white blood cell count, elevated band cell counts [ie, evidence of a "shift to the left"], fever, or highly suggestive radiographic findings) or a confirmed diagnosis of an intra-abdominal infection (presence of pus within the abdominal cavity), and a baseline intra-abdominal culture was obtained from the site of infection. Patients could be enrolled before drainage of the intra-abdominal infection and may have received up to 2 doses of study drug before the baseline cultures were obtained. Patients did not receive more than 1 dose (or combination) of parenteral nonstudy antibacterial drugs after the baseline intra-abdominal cultures were obtained. However, wound irrigation solutions of sterile water or normal saline and topical antiseptics were permitted throughout the course of the study.

### Clinical evaluations

The clinical status of the intra-abdominal infection was assessed at serial visits throughout the study by the presence or absence of the following signs and symptoms: fever; localized or diffuse abdominal wall rigidity or involuntary guarding; abdominal tenderness or pain; ileus or hypoactive bowel sounds; nausea or vomiting. The clinical response to study drug was determined by the investigator. At the test-of-cure visit (14–35 days after therapy), each patient's response was categorized as one of the following: *Cure *– the course of study drug and the initial intervention (operative and/or radiologically guided drainage procedure) resolved the intra-abdominal infectious process; *Failure *– the patient required additional antibacterial therapy other than the study drug, the patient required additional surgical or radiologic intervention to cure the infection, death due to infection occurred after 48 hours of therapy, the patient received an extended course of study drug (ie, >120% of the planned number of doses), or the patient was prematurely discontinued from study drug due to an adverse event (after receiving at least 8 doses in 5 days) and required additional antibiotic therapy or surgical intervention; and *Indeterminate *– the patients was lost to follow-up, or died within 48 hours after the first dose of study drug for any reason, or died after 48 hours because of noninfectious-related reasons (as judged by the investigator).

### Microbiologic evaluations

Baseline aerobic and anaerobic cultures from the primary intra-abdominal site of infection and two sets of blood cultures were obtained within 24 hours of the first dose of study drug. All aerobic and anaerobic bacterial isolates, regardless of the source of cultured material, were identified and tested at a central laboratory (Covance Central Laboratory Services, Inc., Indianapolis, IN, or Geneva, Switzerland) by using a standard procedure approved by the National Committee of Clinical Laboratory Standards (NCCLS) Subcommittee on Antimicrobial Susceptibility Testing. For tigecycline, provisional minimum inhibitory concentration (MIC) breakpoints were used (susceptible ≤2 mg/L; intermediate 4 mg/L; resistant ≥8 mg/L).

Based on the results of the baseline intra-abdominal culture, the susceptibilities of identified organisms, and the clinical outcome of the patient, the investigator also determined the microbiologic response at the patient level and at the isolate level. Microbiologic response by patient was categorized at the test-of-cure visit as eradication, persistence, superinfection (ie, the emergence of a new isolate was documented at the site of infection with worsening signs and symptoms of infection). The microbiologic response for each baseline isolate at the test-of-cure visit was described according to the following definitions: eradication, persistence, or indeterminate. Because many patients did not have follow-up cultures, many microbiologic responses both at the patient and isolate level were categorized as either presumed eradication or presumed persistence.

### Safety/tolerability assessments

All patients who received at least one dose of study drug were evaluated for safety (modified intent-to-treat [mITT] population). Safety was assessed from serial medical history and physical examinations, reports of clinical adverse events, and findings from routine electrocardiograms (ECGs), and serum chemistry, hematology, coagulation, and urinalysis tests. Adverse events were recorded throughout the study period, up to and including the test-of-cure visit. Before unblinding, the investigator categorized the severity of each adverse event and the potential for relationship to study drug. Serious adverse events (ie, those that were life-threatening, led to prolongation of the existing hospitalization, caused persistent or significant disability or incapacity, or death) were also recorded.

### Analysis populations

Several subpopulations of patients were assessed for safety, clinical, and bacteriologic outcomes. Patients who satisfied the inclusion/exclusion criteria were included in the intent-to-treat (ITT) population, whereas the subset of patients who received at least 1 dose of study drug made up the mITT population. Those patients in the mITT population who had clinical evidence of a complicated intra-abdominal infection, by meeting the minimal disease criteria, and had a confirmed baseline isolate made up the microbiological-modified (m-mITT) population. From this latter group, the microbiologically evaluable (ME) population was defined as those who met all inclusion/exclusion criteria; had at least 5 days of therapy; did not receive concomitant antibiotics after the baseline intra-abdominal culture was obtained through the test-of-cure visit; had a test-of-cure visit 14 to 35 days after the first dose of study drug; and had a baseline intra-abdominal culture containing at least one causative isolate that was susceptible to both study drugs. If these criteria were not met at any time during the study, the patient was declared non-evaluable and the outcome of cure/failure/indeterminate was analyzed within the m-mITT population. Patients were considered nonevaluable for inclusion in the ME population if death occurred or if they withdrew from the study <48 hours after the first dose of study drug.

### Statistical analysis

The primary endpoints of the study were clinical response at the test-of-cure visit (14–35 days after therapy) for the m-mITT and ME populations. Secondary analyses included bacteriologic response at the test-of cure visit by patient and isolate, as well as clinical response rates stratified as monomicrobial versus polymicrobial, and by isolate.

Statistical analysis was performed by the Clinical Biostatistics department of Wyeth Research, Collegeville, PA. Categorical baseline demographic and medical variables were analyzed using the Fisher exact test. Continuous variables were compared using a one-way analysis of variance (ANOVA) model with treatment as a factor. Between-group comparisons of adverse events were analyzed by using the Fisher exact test. For laboratory tests, vital signs, and ECG results, within-group changes from baseline were analyzed by using a paired t-test and between-group comparisons were made by using the analysis of covariance, adjusting for baseline value. The difference between treatment groups in the percentage of premature withdrawal from study drug was evaluated by using a 2-sided Fisher exact test.

The noninferiority efficacy of tigecycline compared with imipenem/cilastatin was evaluated for clinical and microbiologic responses by using a 2-sided 95% confidence interval (CI) for the true difference in efficacy (tigecycline minus imipenem/cilastatin) adjusted for the stratification variable APACHE II score and corrected for continuity. Noninferiority was concluded if the lower limit of the 2-sided 95% CI was greater than or equal to -15%. For all subpopulation analyses (eg, monomicrobial versus polymicrobial infection), an adjusted difference between treatment groups with its 95% CI was calculated from a generalized linear model with a binomial probability function and an identity link (SAS^® ^Proc GENMOD). Interaction effects were tested at the 0.10 level of significance. With the planned sample size (n = 788) and an evaluability rate of 50%, the trial had a power of at least 90% to determine the noninferiority of tigecycline compared with imipenem/cilastatin.

## Results

**Figure 1:**
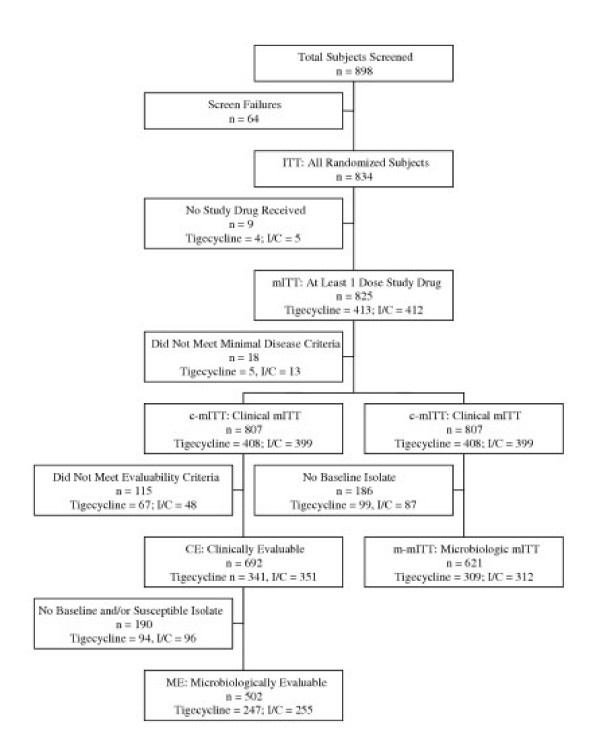
Patient disposition and analysis population.

Eight hundred ninety-eight (898) patients were screened for study participation at 96 sites in 17 countries in the United States, Canada, Europe, Latin America, India, and Asia from November 2002 to August 2004. Of these, 64 patients did not meet protocol requirements (Figure 1). The remaining 834 patients were randomized in a 1:1 ratio to one of the two treatment regimens and represented the ITT population; however, 9 patients never received study drug. Accordingly, 825 patients (413 tigecycline, 412 imipenem/cilastatin) comprised the mITT (safety) population. The majority of the mITT population (98%; 807 of 825) had clinical evidence of a complicated intra-abdominal infection (clinical mITT population). Within this latter cohort, 692 patients were clinically evaluable (clinically evaluable [CE] population). One hundred thirty three (133; 16.1%) mITT patients (72 tigecycline, 61 imipenem/cilastatin) were not included in the CE population for the following primary reasons (patients could have been excluded for more than one reason): no clinical evaluation at the test-of-cure visit (n = 47); entry criteria not met (n = 28); blind broken (n = 22); and received more than 1 dose of a nonstudy antibiotic after pretherapy culture (n = 12). From the mITT population, 621 of 825 (75%) patients had a pretherapy isolate isolated and comprised the m-mITT population. A total of 502 m-mITT patients (247 tigecycline, 255 imipenem/cilastatin) met both clinical evaluability criteria and had a pretherapy isolate isolated from an intra-abdominal source (ME population).

### Demographic/baseline medical characteristics

The demographic characteristics for the 502 ME patients were comparable between the two treatment groups (Table 1). The study population was of mixed racial/ethnic background with whites (41.8%) and Hispanics (19.5%) represented most often. There was a predominance of men (67.5%) and the mean age of enrolled patients was 43 years old. Complicated appendicitis (59%) was the most common intra-abdominal infection diagnosis, followed by perforated intestine (8.8%) and gastric/duodenal ulcer (4.6%). No significant differences between the treatment groups were observed in the number or types of infections diagnosed at baseline. The severity of intra-abdominal illness was similar in each treatment group (mean APACHE II score was ~5.7).

**Table 1:** Demographic and baseline medical characteristics (ME population)

	Tigecycline N = 247	Imipenem/Cilastatin N = 255
Mean ± SD age, years	42.9 ± 18.0	43.1 ± 17.6
Sex, n (%) male	173 (70.0)	166 (65.1)
Ethnic origin, n (%)		
White	104 (42.1)	106 (41.6)
Black	16 (6.5)	25 (9.8)
Asian	30 (12.1)	30 (11.8)
Hispanic	54 (21.9)	44 (17.3)
Other	43 (17.4)	50 (19.6)
Mean ± SD weight, kg	70.3 ± 15.7	69.3 ± 15.9
Mean ± SD creatinine clearance, mL/min	94.2 ± 35.3	94.3 ± 34.1
Mean ± SD therapy duration, days	8.1 ± 2.8	7.9 ± 2.7
Mean APACHE II score	5.6	5.5
Primary intra-abdominal diagnosis, n (%)		
Complicated appendicitis	152 (61.5)	145 (56.9)
Perforation of intestine	21 (8.5)	23 (9.0)
Complicated diverticulitis	17 (6.9)	25 (9.8)
Intra-abdominal abscess	17 (6.9)	17 (6.7)
Peritonitis	14 (5.7)	16 (6.3)
Gastric/duodenal perforation	13 (5.3)	10 (3.9)
Complicated cholecystitis	12 (4.9)	16 (6.3)
Other*	1 (0.4)	3 (1.2)

*Other diagnoses included infected hematoma, pelvic inflammatory disease, acute abdomen subocclusion, acute inflammatory abdomen, disease pelvic infectious, tubo-ovarian abscess, right tubal abscess, infected left subphrenic hematoma.

### Clinical efficacy

For the ME population, clinical cure rates were 80.6% for tigecycline and 82.4% for imipenem/cilastatin (95% CI -9.0, 5.4; Table 2). Corresponding clinical cure rates for the m-mITT population were 73.5% and 78.2% (95% CI -11.8, 2.3), respectively. For both the ME and m-mITT populations, tigecycline was efficacious and statistically noninferior to imipenem/cilastatin. Multiple subgroup analyses of clinical responses (eg, age, sex, race, geographic location) found consistently efficacious clinical responses between the treatment groups. No significant treatment differences in clinical response were observed between the two treatment groups when patients were stratified by the number of isolated baseline isolates (Table 2). For the ME population, tigecycline had a 89.8% clinical cure rate at the test-of-cure visit for monomicrobial infections and a 75.3% clinical cure rate for polymicrobial infections. Similar rates were observed for recipients of imipenem/cilastatin (88.5% and 78.1%, respectively).

**Table 2:** Clinical cure rates at test-of-cure visit

	Tigecycline		Imipenem/cilastatin		Difference Tigecycline-Imipenem/cilastatin	Test for Noninferiority	Test for Differences
	
Population	N	% (95% CI)	N	% (95% CI)	% (95% CI)	*P *value	
CE	282/341	82.7 (78.3, 86.6)	295/351	84.0 (79.8, 87.7)	-1 (-7.2, 4.5)	<0.0001	0.70
Overall							-1 (-6.9, 4.2)*
c-mITT	303/408	74.3 (69.7, 78.4)	317/399	79.4 (75.1, 83.3)	-5 (-11.2, 0.0)	<0.0001	0.00
Overall							-5 (-11.0, 0.0)
ME	199/247	80.6 (75.1, 85.3)	210/255	82.4 (77.1, 86.8)	-1.8 (-9.0, 5.4)	0.0001	0.6892
Monomicrobial	80/89	89.9 (81.7, 95.3)	92/104	88.5 (80.7, 93.9)	1.4 (-8.7, 11.0)		
Polymicrobial	119/158	75.3 (67.8, 81.8)	118/151	78.1 (70.7, 84.5)	-2.8 (-12.6, 7.1)		
Overall							-1.7 (-8.4, 5.1)*
m-mITT	227/309	73.5 (68.2, 78.3)	244/312	78.2 (73.2, 82.7)	-4.7 (-11.8, 2.3)	0.0019	0.1976
Monomicrobial	96/121	79.3 (71.0, 86.2)	109/128	85.2 (77.8, 90.8)	-5.8 (-15.9, 4.3)		
Polymicrobial	131/188	69.7 (62.6, 76.2)	135/184	73.4 (66.4, 79.6)	-3.7 (-13.1, 5.9)		
Overall							-4.3 (-11.0, 2.5)*

*Adjusted difference and its 95%CI are calculated from a generalized linear model with a binomial probability function and an identity link.

For complicated appendicitis, the most frequent diagnosis, clinical cure rates at the test-of-cure visit was 84.2% for tigecycline and 86.2% for imipenem/cilastatin (Table 3). In both treatment groups, lower clinical cure rates (≤72%) were observed in patients who had intra-abdominal abscess, complicated diverticulitis, or intestinal perforation (Table 3). Overall, there were no significant differences in clinical cure rates between tigecycline and imipenem/cilastatin based on primary intra-abdominal diagnosis. A total of 14 tigecycline- and 27 imipenem/cilastatin-treated patients in the ME population had a positive pretherapy blood culture. Clinical cure in patients with bacteremia was reported for 71.4% of tigecycline and 74.1% of imipenen/cilastatin recipients.

**Table 3:** Clinical cure rate by baseline diagnosis (ME population) at test-of-cure visit

	Tigecycline		Imipenem/cilastatin		Difference Tigecycline-Imipenem/cilastatin
	
Clinical Diagnosis	N	% (95% CI)	N	% (95% CI)	% (95% CI)
Complicated appendicitis	128/152	84.2 (77.4, 89.6)	125/145	86.2 (79.5, 91.4)	-2.0 (-10.6, 6.7)
Perforation of the intestines	13/21	61.9 (38.4, 81.9)	15/23	65.2 (42.7, 83.6)	-3.3 (-32.4, 26.2)
Complicated diverticulitis	12/17	70.6 (44.0, 89.7)	18/25	72.0 (50.6, 87.9)	-1.4 (-32.0, 26.7)
Intra-abdominal abscess	11/17	64.7 (38.3, 85.8)	12/17	70.6 (44.0, 89.7)	-5.9 (-37.6, 27.4)
Peritonitis	12/14	85.7 (57.2, 98.2)	15/16	93.8 (69.8, 99.8)	-8.0 (-38.2, 20.5)
Complicated cholecystitis	11/12	91.7 (61.5, 99.8)	14/16	87.5 (61.7, 98.4)	4.2 (-29.4, 32.4)
Gastric and abdominal perforations	11/13	84.6 (54.6, 98.1)	10/10	100.0 (69.2, 100.0)	-15.4 (-46.3, 21.3)
Other	1/1	100.0 (2.5, 100.0)	1/3	33.3 (0.8, 90.6)	66.7 (-42.3, 98.2)
Concomitant bacteremia	10/14	71.4 (41.9, 91.6)	20/27	74.1 (53.7, 88.9)	-2.6 (-35.3, 25.4)

### Microbiologic efficacy

For the ME population, eradication of intra-abdominal isolates at the patient level was reported for 80.6% of tigecycline- and 82.4% of imipenem/cilastatin-treated patients (95% CI -9.0, 5.4), indicating that tigecycline was efficacious and statistically noninferior to imipenem/cilastatin (Table 4). No significant differences between the treatment groups were found when eradication rates were stratified by monomicrobial versus polymicrobial infection (Table 4).

**Table 4:** Microbiologic response at the patient level (ME Population) at test-of-cure visit

	Tigecycline		Imipenem/cilastatin		Difference Tigecycline-Imipenem/cilastatin	Test for Noninferiority	Test for Differences
	
Response	N	% (95% CI)	N	% (95% CI)	% (95% CI)	*P *value	
Eradication	199/247	80.6 (75.1, 85.3)	210/255	82.4 (77.1, 86.8)	-1.8 (-9.0, 5.4)	0.0001	0.6892
							
Persistence	39/247	15.8 (11.5, 20.9)	42/255	16.5 (12.1, 21.6)			
							
Documented	4/39	10.3 (2.9, 24.2)	1/42	2.4 (0.1, 12.6)			
Presumed	35/39	89.7 (75.8, 97.1)	41/42	97.6 (87.4, 99.9)			
Superinfection	9/247	3.6 (1.7, 6.8)	3/255	1.2 (0.2, 3.4)			
							
Overall					-1.7 (-8.4, 5.1)*		

*Adjusted difference and its 95%CI are calculated from a generalized linear model with a binomial probability function and an identity link.

Generally, eradication rates at the test-of-cure visit for the most commonly isolated intra-abdominal isolates were similar between the two treatment groups (Table 5). For *E. coli*, the most commonly isolated aerobe, eradication rates were 80.4% for tigecycline versus 83.5% for imipenem/cilastatin. Corresponding eradication rates for *Klebsiella *spp, the second most frequently isolated gram-negative aerobe, were 87.1% and 85.7%, respectively. A total of 6 ESBL-producing *E. coli *and 7 ESBL-producing *K. pneumoniae *isolates were identified pretherapy. The majority of these isolates were eradicated by tigecycline: 83% (5/6) and 71% (5/7), respectively. Eradication rates for *Bacteroides fragilis *were 69.8% for tigecycline and 72.5% for imipenem/cilastatin.

**Table 5:** Microbiologic response at the isolate level: selected baseline isolates at test-of-cure visit (ME population)

	Tigecycline			Imipenem/cilastatin		
	
Isolate	N	MIC_90_	% (95% CI)	N	MIC_90_	% (95% CI)
*Bacteroides fragilis*	30/43	2.0	69.8 (53.9, 82.8)	29/40	0.5	72.5 (56.1, 85.4)
*Citrobacter *spp.	13/15	1.0	86.7 (59.5, 98.3)	5/7	0.5	71.4 (29.0, 96.3)
*Clostridium *spp.	16/19	1.0	84.2 (60.4, 96.6)	14/18	2.0	77.8 (52.4, 93.6)
*Enterobacter *spp.	6/8	1.0	75.0 (34.9, 96.8)	5/10	1.0	50.0 (18.7, 81.3)
*Enterococcus faecalis *(non-VRE)	10/16	0.25	62.5 (35.4, 84.8)	9/18	4.0	50.0 (26.0, 74.0)
*Escherichia coli*	135/168	0.5	80.4 (73.5, 86.1)	152/182	0.25	83.5 (77.3, 88.6)
*Fusobacterium *spp.	3/5	0.25	60.0 (14.7, 94.7)	6/7	0.25	85.7 (42.1, 99.6)
*Klebsiella *spp.	27/31	1.0	87.1 (70.2, 96.4)	36/42	0.25	85.7 (71.5, 94.6)
*Peptostreptococcus *spp.	6/10	0.12	60.0 (26.2, 87.8)	5/8	0.25	62.5 (24.5, 91.5)
*Proteus *spp.	5/10	4.0	50.0 (18.7, 81.3)	3/3	4.0	100.0 (29.2, 100.0)
*Pseudomonas aeruginosa*	13/18	32.0	72.2 (46.5, 90.3)	19/21	2.0	90.5 (69.6, 98.8)
*Staphylococcus aureus *(MRSA)	1/2	NA	50.0 (1.3, 98.7)	0/1	NA	0.0 (0.0, 97.5)
*S. aureus *(non-MRSA)	7/8	0.25	87.5 (47.3, 99.7)	3/4	0.12	75.0 (19.4, 99.4)
*Streptococcus *spp.	63/81	0.12	77.8 (67.2, 86.3)	46/67	0.12	68.7 (56.2, 79.4)

MRSA = methicillin-resistant *Staphylococcus aureus; *VRE = vancomycin-resistant enterococci.

NA = MIC_90 _values are not valid if the number of isolates is less than 10.

Pretherapy in vitro activity against baseline isolates for tigecycline and imipenem/cilastatin are shown in Table 6. The mean MIC_90 _for tigecycline against the most commonly isolated aerobes and anaerobes was ≤2.0 mg/L. No pretherapy isolates displayed resistance to tigecycline based on the provisional breakpoints used. Bacterial susceptibilities to tigecycline appeared to be consistent with clinical responses.

**Table 6:** MIC range, and MIC_50 _and MIC_90 _values of selected primary baseline isolates (ME population)

		Tigecycline			Imipenem/Cilastatin		
		
Isolate	n	MIC range	MIC_50_	MIC_90_	MIC range	MIC_50_	MIC_90_
*Bacteroides fragilis*	83	0.06–16.0	1.0	2.0	0.12–4.0	0.25	0.5
*Clostridium perfringens*	12	0.06–2.0	1.0	2.0	0.12–0.25	0.12	0.25
*Enterococcus faecalis *(non-VRE)	32	0.06–0.25	0.12	0.25	1.0–4.0	1.0	4.0
*Escherichia coli*	350	0.06–1.0	0.25	0.50	0.12–1.0	0.12	0.25
*Klebsiella pneumoniae*	58	0.25–2.0	0.50	1.00	0.12–0.50	0.25	0.25
*Pseudomonas aeruginosa*	39	8.0–32.0	16.0	32.0	0.25–4.0	1.0	2.0
*Staphylococcus aureus *(MRSA)	3	0.12–0.25	NA	NA	0.12–32.0	NA	NA
*S. aureus *(non-MRSA)	12	0.12–0.50	0.25	0.25	0.12–0.12	0.12	0.12

NA = MIC_50 _and MIC_90 _values are not valid if the number of isolates is less than 10.

MRSA = methicillin-resistant *Staphylococcus aureus.*

VRE = vancomycin-resistant enterococci.

### Safety and tolerability

Data from all patients in the mITT population (n = 825) were analyzed for safety. The m-ITT population received a median of 7 days of tigecycline or imipenem/cilastatin treatment. Regardless of study drug causality, the frequency and distribution of treatment-emergent adverse events occurring in at least 3% of patients in either treatment group were similar to those observed in the imipenem/cilastatin treatment group. The majority of these adverse events were related to study medication (56%) and were mild to moderate in intensity (94%). Digestive system (56.9% vs 49.8%, *P *= 0.043), nausea (31.0% tigecycline, 24.8% imipenem/cilastatin; *P *= 0.052), vomiting (25.7% tigecycline, 19.4% imipenem/cilastatin; *P *= 0.037), and diarrhea (21.3% tigecycline, 18.9% imipenem/cilastatin; *P *= 0.435) were the most frequently reported adverse events in both treatment groups. The majority of patients in both treatment groups experienced mild to moderate nausea and/or vomiting (94%). There was no significant difference between the treatment groups in the number of patients who required antiemetic therapy for nausea and/or vomiting. No tigecycline-treated patient has a positive *Clostridium difficile *toxin assay, nor developed *C. difficile *associated diarrhea.

In the tigecycline group, infections (13.6% vs 7.5%, P = 0.006), hypoproteinemia (8.0% vs 4.1%, *P *= 0.028), and dyspnea (6.8% vs 2.9%, *P *= 0.014) were statistically higher than in the imipenem/cilastatin treatment group. The difference in infection rates between the treatment groups was primarily due to the development of secondary wound infections. No apparent trends or risk factors were identified in the development of secondary wound infections in either treatment group.

One hundred forty-five (145) patients had one or more serious adverse events during the study (81 [19.6%] tigecycline, 64 [15.5%] imipenem/cilastatin) (*P *= 0.143). The most frequently reported serious adverse events were abnormal healing (14 tigecycline, 6 imipenem/cilastatin), abscess (10 tigecycline, 8 imipenem/cilastatin), and infection (10 tigecycline, 9 imipenem/cilastatin). Significantly more patients treated with tigecycline (6 [1.5%]) versus none treated with imipenem/cilastatin reported pneumonia as a serious adverse event (*P *= 0.031).

Adverse events were the primary reason for early withdrawal of study drug. A total of 27 (6.5%) tigecycline- and 15 (3.6%) imipenem/cilastatin-treated patients discontinued treatment prematurely because of an adverse event (*P *= 0.080). A total of 10 (2.5%) tigecycline- and 4 (1.0%) imipenem/cilastatin-treated patients stopped therapy prematurely secondary to either nausea (6 tigecycline, 2 imipenem/cilastatin) and/or vomiting (4 tigecycline, 2 imipenem/cilastatin). There were no significant differences between treatment groups in any single adverse event leading to the discontinuation of study drug.

Twenty-nine (29) patients died during the study: 17 patients in the tigecycline group and 12 patients in the imipenem/cilastatin treatment group. Only two of the deaths, both in the tigecycline group, were considered by the investigators to be possibly related to study drug secondary to treatment failure. The first patient was a 78 year old female who received tigecycline for one week. Two days following discontinuation of therapy the patient developed septic shock; she died one day later. The second patient, a 23 year old female, presented with sepsis and received 3 days of tigecycline therapy. On day 3 she was found to have pneumonia and progressed to multiple organ failure with sepsis and died the same day.

Few clinically important or unexpected changes in any routine hematologic or serum chemistry tests, vital signs, or ECG data were associated with the use of tigecycline or imipenem/cilastatin. However, significantly more patients treated with imipenem/cilastatin (312/410, 76.1%) than those treated with tigecycline (275/408, 67.4%) had 1 or more laboratory findings of potential clinical importance (*P *= 0.007). Imipenem/cilastatin-treated patients had significantly lower serum potassium (≤3 mmol/L; *P *= 0.004), phosphorus values (≤ 0.8 mmol/L; *P *< 0.001), and lymphocytes values (≤0.6 cells × 10^9^/L; *P *< 0.001). Yet, significantly more patients treated with tigecycline than those treated with imipenem/cilastatin had clinically significant hypoproteinemia (≤35 g/L; *P *= 0.001). No significant changes in QTc interval were observed in either treatment group at any time point.

## Discussion

This large trial demonstrated that tigecycline (100 mg initial dose, followed by 50 mg q12 hours) is effective for the treatment of hospitalized adult patients with complicated intra-abdominal infections. For patients with proven bacterial infections, clinical cure rates were 80.6% for tigecycline versus 82.4% for imipenem/cilastatin at the test-of-cure visit, demonstrating that tigecycline met the statistical criteria for noninferiority compared with the carbapenem regimen. We also observed that tigecycline's clinical efficacy was similarly effective in patients who had either monomicrobial versus polymicrobial infection, as well as across the variety of anatomical infections encountered. While many previous studies have reported a higher percentage of polymicrobial infection from intra-abdominal sites, the lower rates seen with tigecycline may be explained by a larger proportion of patients with appendicitis as the source of infection. Overall, the efficacy of tigecycline was consistent among all predefined populations analyzed (m-mITT, c-mITT, CE) and consistent across different species of infecting bacteria.

This large study extends the findings of two other studies that evaluated tigecycline's efficacy in the treatment of complicated intra-abdominal infections. In a small, open-label, phase 2 tigecycline trial of 66 hospitalized patients with primarily perforated appendicitis, cure rates at the test-of-cure visit and end-of-treatment visit were 67% and 76%, respectively [17]. A similarly designed phase 3 trial reported comparable clinical cure rates for the m-mITT cohort of 86.6% (279/322) for tigecycline compared with 84.6% (270/319) for imipenem/cilastatin therapy [18].

The current trial demonstrated that tigecycline was effective at eradicating commonly encountered aerobic and anaerobic intestinal bacteria. Overall eradication rates were nearly identical in the two treatment groups: 80.6% after tigecycline therapy compared with 82.4% in the imipenem/cilastatin group. More than 80% of *E. coli *and *Klebsiella *spp. (the two most frequently isolated gram-negative aerobes) were eradicated by tigecycline, followed by 78% of *Streptococcus *spp, and 70% of *B. fragilis*. Comparable eradication rates were observed following imipenem/cilastatin therapy, further establishing that tigecycline was at least as effective as the standard carbapenem regimen. These data support in vitro observations that tigecycline has broad-spectrum activity against common isolates found in intra-abdominal infections [8-16]. While the etiologic role of *P. aeruginosa *remains unclear in patients with community-acquired intra-abdominal infections, tigecycline lacks reliable in vitro activity against this organism [8,9,11,13] despite a 72% eradication rate in this study.

Because few resistant isolates were isolated in the current trial, we could not conclusively establish the in vivo effectiveness of tigecycline against organisms that typically convey resistance (eg, *E. faecalis*, methicillin-sensitive and -resistant *S. aureus*, ESBL-producing *Enterobacter *spp.). However, tigecycline successfully eradicated the majority (77%) of the 13 ESBL-producing *E. coli *and *K. pneumoniae *that were recovered from patients with cIAI. These limited data confirm tigecycline's documented in vitro activity against many gram-positive and gram-negative bacterial isolates that typically are resistant [11,16,19].

Both tigecycline and imipenem/cilastatin were well tolerated in the current trial, with a similar frequency and distribution of treatment-emergent adverse events. Nausea, vomiting, and diarrhea were the most frequently reported adverse events in both the tigecycline and imipenem/cilastatin treatment groups. Although the individual adverse events of nausea and vomiting occurred at higher rates after tigecycline compared with imipenem/cilastatin therapy, only the incidence of vomiting was found to be significantly higher in tigecycline recipients. Furthermore, the majority of nausea/vomiting events in both treatment groups were of mild to moderate intensity (94%). Supporting this fact, these gastrointestinal adverse events rarely led to early discontinuation of therapy in either treatment group (<2%) and there was no difference in the number of patients requiring interventional antiemetic therapy between the tigecycline and imipenem/cilastatin groups. It is also noteworthy that tigecycline monotherapy was not associated with the development of *C. difficile *diarrhea. These findings support previous safety data from phase 2 and 3 studies [20-25].

## Conclusion

Tigecycline is an effective and well-tolerated monotherapy option for the treatment of patients with complicated intra-abdominal infections, with comparable efficacy to imipenem/cilastatin. Because of the rising rates of antibiotic-resistant bacteria, both in the community and hospital settings, there remains a need for new antibiotic options. According, tigecycline is a promising new monotherapy when empiric coverage is needed against both gram-positive and non-pseudomonal gram-negative bacteria, including improved in vitro activity against certain resistant isolates.

## List of abbreviations

AIDS – acquired immunodeficiency syndrome

ALT – alanine aminotransferase

ANOVA – analysis of variance

APACHE – Acute Physiologic and Chronic Health Evaluation

AST – aspartate aminotransferase

CE – clinically evaluable

cIAI – complicated intra-abdominal infections

CI – confidence interval

c-mITT – clinical modified intent-to-treat

ECG – electrocardiograms

ESBL – extended spectrum beta-lactamases

IDSA – Infectious Diseases Society of America

IMI/CIS – imipenem/cilastatin

ITT – intent-to-treat

IV – intravenous

ME – microbiologically evaluable

MIC – minimum inhibitory concentration

m-mITT – microbiologically modified intent-to-treat

MRSA – methicillin-resistant *Staphylococcus aureus*

NCCLS – National Committee of Clinical Laboratory Standards

q12h – every 12 hours

SD – standard deviation

TOC – test-of-cure

ULN – upper limit of normal

VRE – Vancomycin-Resistant Enterococci

## Competing interests

Financial competing interests: Drs. Oliva, Rekha, Yellin, Paternak, and Campos are investigators for this tigecycline study sponsored by Wyeth. Dr. Oliva is an investigator for a clinical trial sponsored by Roche. Drs. Rose, Babinchak, Ellis-Grosse, and Loh are employees of Wyeth. None of the authors have non-financial competing interests to disclose.

## Authors' contributions

The first five authors were investigators in the clinical trial and enrolled the highest number of evaluable patients (MEO, AR, AY, JP, MC). Authors GMR, TB, EE-G, EL made substantial contributions to the conception and design of the study. All authors made substantial contributions acquisition of data and analysis and interpretation of data. Each author (MEO, AR, AY, JP, MC, GMR, TB, EE-G, EL) was involved in critically revising the paper for intellectual content and has given final approval of this version to be published.

## Pre-publication history

The pre-publication history for this paper can be accessed here:


